# SA-DETR: Lightweight Structure-Aware Defect Detection Model for Sustainable Low-Carbon Steel Manufacturing Based on RT-DETR-L

**DOI:** 10.3390/s26196075

**Published:** 2026-09-25

**Authors:** Peifeng Liang, Xinyue Wu

**Affiliations:** School of Computer Engineering, Jiangsu University of Technology, Changzhou 213000, China; 2024655156@smail.jsut.edu.cn

**Keywords:** RT-DETR, steel surface defect, lightweight, sustainable

## Abstract

Steel manufacturing is a typical high-energy and high-carbon industry. Undetected tiny surface defects such as scratches and crazing lead to massive waste of steel raw materials and extra energy consumption in subsequent processing, while heavy detection models may increase the computational energy demand of on-site inspection systems. The purpose of this study is to develop a lightweight defect detection model that improves the recognition of weak-semantic and structurally distinctive surface defects while reducing computational cost for industrial deployment. To achieve this objective, we propose SA-DETR, a lightweight structure-aware defect detection model built on RT-DETR-L. Three targeted modules are introduced: O-EHGBlock retains fine-grained structural cues of micro-defects with reduced computation; Adaptive Structure-aware Query Selection (ASQS) supplements semantic query ranking with local structural information to improve the detection of weak-semantic defects; and lightweight StarC3 replaces redundant fusion blocks to cut computational overhead. Evaluated on NEU-DET and PCB datasets, SA-DETR boosts mAP@0.5 by 4.5 percentage points over RT-DETR-L, while reducing parameters by 36.0% and GFLOPs by 46.5%, with inference speed lifted to 103.0 FPS. These results demonstrate that SA-DETR provides a favorable trade-off between detection accuracy and computational efficiency, indicating its potential for resource-constrained industrial surface inspection and sustainable steel quality control.

## 1. Introduction

Steel is a fundamental material used in infrastructure construction, automobile manufacturing, aerospace engineering, and high-end equipment production. Its surface quality directly affects the service performance, structural reliability, and lifetime of steel products. During rolling, cooling, pickling, and transportation, various surface defects may occur, including crazing, inclusions, patches, pitted surfaces, rolled-in scales, and scratches. As an energy-intensive and high-carbon industrial sector, steel production poses prominent challenges to green and sustainable manufacturing. Unidentified micro-defects such as fine scratches and crazing flow into subsequent rolling, welding, and coating procedures, causing large batches of finished steel products to be scrapped. This unnecessary waste consumes substantial iron ore, water, and fossil fuels, aggravating industrial carbon emissions. Meanwhile, traditional manual inspection relies on intensive human labor with unstable accuracy, whereas conventional deep learning detectors may increase the computational energy demand of real-time on-site inspection systems. Therefore, the purpose of this study is to develop a lightweight detection model that improves the recognition of tiny steel surface defects with weak semantic responses while reducing computational cost for industrial deployment [1].

Traditional steel surface defect inspection mainly relies on manual visual inspection and machine vision methods based on handcrafted features [2]. However, manual inspection is time-consuming and susceptible to subjective fatigue, resulting in unstable detection accuracy. Meanwhile, traditional machine vision approaches based on handcrafted feature engineering heavily depend on expert experience, and it is difficult to achieve satisfactory detection performance when facing complex surface textures, uneven illumination, and diverse defect types. In recent years, deep learning-based detection algorithms have shown powerful automatic feature extraction capabilities, providing a new technical route for industrial steel surface defect detection.

Convolutional neural network-based object detectors, including Faster R-CNN [3], SSD [4], and the YOLO family [5,6], are capable of learning discriminative defect features in an end-to-end manner, and they have obtained competitive performance on steel surface defect detection tasks [7,8]. Nonetheless, mainstream CNN detection architectures depend on dense prediction, region proposal generation, or non-maximum suppression. Consequently, the detection workflow still involves manually engineered modules and post-processing steps. When confronting densely distributed defects, drastic scale changes, and intricate background textures, mutual competition among candidate bounding boxes together with post-processing may introduce false positives and missed targets [7]. Furthermore, successive downsampling and local convolution suppress edge and texture cues of miniature defects, which impairs the model’s capability to capture fine-grained structural details [8].

The advancement of Transformers [9] in computer vision has pioneered a new paradigm for object detection. DETR [10] formulates object detection as a set prediction task and leverages bipartite matching to enable end-to-end prediction without the conventional non-maximum suppression pipeline. A series of optimized variants, including Deformable DETR [11], DAB-DETR [12], DINO [13], and RT-DETR [14], have been proposed to mitigate the drawbacks of vanilla DETR, namely slow training convergence, high computational complexity, and inferior performance for small-object detection. As a representative real-time variant, RT-DETR adopts an efficient hybrid encoder and a query selection mechanism, achieving a favorable trade-off between detection accuracy and inference speed. Accordingly, it serves as a promising end-to-end framework for industrial surface defect detection.

Although RT-DETR has achieved favorable performance in general object detection tasks [14], several challenges remain when directly applying this model to the detection of tiny steel surface defects [15]. First, defects such as scratches, crazing, and inclusions only occupy a small number of pixels, and their discriminative features are mainly reflected in local edges, texture variations, and directional structures. Continuous downsampling and dense spatial convolution operations in the original backbone network tend to weaken such fine-grained features [16]. Although lightweight operations can reduce computational consumption, they may further suppress the local structural responses of tiny defects. Therefore, the lightweight backbone network needs to reduce redundant calculations while retaining and enhancing elongated edge features and local texture information.

Second, RT-DETR initializes object queries mainly based on the semantic scores output by the encoder classification head and selects feature positions with the Top-K confidence scores as initial queries [14]. For conventional detection targets, deep semantic responses can usually provide reliable regional cues. However, tiny steel surface defects generally suffer from low contrast, small spatial coverage, and weak semantic features. Their classification response values may be lower than those generated by complex background regions. Some defective regions exhibit distinct edges, abrupt grayscale changes, and local geometric variations, yet they are still likely to be excluded from the Top-K query set owing to insufficient semantic scores [17]. Accordingly, the query selection strategy merely dependent on semantic information fails to realize effective query initialization for tiny defects with weak semantic responses.

Existing DETR variants have introduced saliency information into query design from different perspectives. SAP-DETR employs salient points as query-specific spatial priors and progressively transforms them into object instances, primarily improving spatial initialization and convergence [18]. Salience DETR uses hierarchical salience filtering, scale-independent salience supervision, and query refinement to alleviate scale bias and suppress redundant encoder tokens [19]. Although these methods demonstrate the effectiveness of saliency-aware query design, they mainly focus on generic spatial-prior initialization, scale bias, or efficient token filtering. In contrast, the proposed ASQS is designed for industrial defects whose semantic responses are weak but whose local structural variations remain distinctive. It directly extracts lightweight structural cues from encoder features to complement the original semantic Top-K ranking of RT-DETR and subsequently modulates the selected query representations using structural responses. Thus, structural information contributes to both candidate selection and query representation, which constitutes the main distinction between ASQS and existing saliency-aware query mechanisms.

Third, the cross-scale feature fusion network of RT-DETR adopts convolution operations on multiple feature branches to strengthen the feature representation of targets at different scales [14]. The original feature fusion module supports sufficient feature interaction. Nevertheless, repeated deployment of this module in multiscale paths increases model parameters and computational overhead, which impedes the deployment of detection models on industrial equipment with limited computing resources [20]. Moreover, existing lightweight Transformer detectors are not specifically optimized to preserve the sparse structural responses of tiny steel defects during backbone extraction, query initialization, and cross-scale feature fusion [21]. In this context, reducing the complexity of feature fusion modules while maintaining effective cross-scale feature interaction is another key research issue.

To tackle the aforementioned issues, this paper proposes SA-DETR, a lightweight structure-aware steel surface defect detection model based on RT-DETR-L. The model design adheres to three core principles: fine-grained structure preservation, structure-aware query initialization, and efficient cross-scale feature fusion. Corresponding targeted optimizations are implemented in the backbone network, query selection strategy, and cross-scale feature fusion module to comprehensively address the inherent deficiencies of the original RT-DETR model in tiny steel defect detection and industrial deployment. Rather than pursuing the highest detection accuracy through increased model scale, SA-DETR aims to achieve a favorable balance between defect detection accuracy and computational efficiency.

Specifically, three dedicated modules are designed to implement the above optimization paradigms. First, an optimized efficient hierarchical ghost block (O-EHGBlock) is proposed for high-efficiency hierarchical feature extraction. This module integrates multiscale partial convolution, a lightweight structural enhancement branch, and a fused coordinate attention mechanism. Through this design, redundant spatial computations are reduced, while the representation of local edges, subtle textures, and directional structural features of tiny defects is enhanced. Second, an adaptive structure-aware query selection (ASQS) mechanism is developed, which combines encoder-derived semantic responses with lightweight local structural cues. Different from the original semantics-only query initialization scheme, the proposed ASQS mechanism assigns higher query priority to defect regions with prominent structural variations but weak semantic responses. Furthermore, a dynamic structural gating unit is embedded to strengthen the structural feature representation of the selected queries. Consequently, ASQS uses structural information at two complementary levels: recalibrating which candidate locations are selected and enhancing how the selected queries are represented. Third, a lightweight StarC3 feature fusion module is constructed based on star transformation operations. Equipped with dual-branch feature propagation, element-wise feature multiplication, and a LayerScale adaptive scaling mechanism, the proposed module achieves efficient nonlinear cross-scale feature interaction. This design effectively reduces overall model complexity while maintaining effective cross-scale feature fusion.

The main contributions of this paper are summarized as follows:

1. A lightweight backbone module termed O-EHGBlock is designed. It reduces redundant computational overhead while enhancing the fine-grained structural representation capability for tiny steel surface defects, achieving a favorable trade-off between feature extraction performance and model lightweightness.
2. An adaptive structure-aware query selection mechanism is proposed. By using local structural cues to complement semantic query ranking and adaptively modulate the selected query representations, ASQS improves query initialization for defects with weak semantic responses and distinctive structural variations.
3. A lightweight StarC3 feature fusion module is developed. The module realizes effective cross-scale feature interaction with fewer parameters and computations, lowering the deployment threshold of detection models on resource-limited industrial devices.
4. Comparative and ablation experiments on the NEU-DET and PCB datasets validate the effectiveness and complementarity of the proposed modules. The results demonstrate that SA-DETR achieves a favorable balance among detection accuracy, parameter count, computational complexity, and inference speed.

## 2. Related Work

Lightweight network design is an important requirement for deploying steel surface defect detectors on resource-constrained industrial platforms. Current lightweight modeling paradigms, including partial convolution, depthwise separable convolution, and group convolution, can reduce model parameters and computational cost by limiting redundant channel and spatial operations [22]. These lightweight convolution strategies have been widely adopted in real-time defect detection systems to meet edge deployment demands [20]. However, computational savings may be accompanied by weakened fine-grained feature representation. For tiny steel defects whose discriminative information is concentrated in subtle edges and local directional textures, excessive feature simplification may attenuate informative defect cues [23]. Therefore, balancing computational efficiency and fine-grained structural feature retention has become a core challenge for lightweight micro-defect detection backbones [24].

Cross-scale feature fusion is indispensable for capturing multiscale defect features in complex industrial steel backgrounds. Existing fusion architectures commonly employ sequential top-down and bottom-up propagation to facilitate cross-level feature interaction [25]. Nevertheless, conventional dense fusion paradigms involve repeated convolutional operations and parameter accumulation, resulting in high computational costs and low inference efficiency that limit their applicability to resource-constrained industrial platforms [15]. In particular, for small steel defects characterized by scale variation, sparse spatial distribution, and weak responses, repeated cross-scale transformations may introduce feature redundancy and further attenuate weak defect representations [26]. Developing lightweight, defect-oriented cross-scale fusion mechanisms is thus critical for practical steel inspection applications [27].

Query initialization directly influences feature matching and subsequent decoding performance in transformer-based detectors [14]. The original RT-DETR ranks encoder tokens according to the classification logits produced by the encoder head and selects the Top-K tokens as initial object queries. Although this semantic ranking strategy is effective for objects with strong category-level responses, it may be suboptimal for tiny steel surface defects. Such defects often exhibit low contrast and limited spatial coverage, and their discriminative information may be expressed more strongly through local edges, grayscale discontinuities, and geometric variations than through high-level semantic responses [17]. Consequently, the semantics-only query ranking strategy may assign insufficient priority to structurally distinctive but semantically weak defect regions, causing them to be excluded from the Top-K candidate set and increasing the risk of missed detections. This limitation motivates the use of local structural evidence as a complement to semantic confidence during query initialization.

Existing DETR variants have investigated saliency-aware query design from different perspectives. SAP-DETR initializes query-specific reference points from salient locations and progressively transforms these point representations into object instances, primarily improving spatial initialization and convergence [18]. Salience DETR introduces hierarchical salience filtering together with scale-independent salience supervision and query refinement to alleviate scale bias, reduce redundant computation, and stabilize two-stage query initialization [19]. These studies demonstrate the value of saliency information for DETR-based detection, but their primary objectives are salient-point-based spatial initialization, efficient token filtering, and stable two-stage query generation.

In contrast, ASQS is designed for industrial defects that exhibit weak semantic responses but distinctive local structural variations. Its methodological distinction lies in introducing structural evidence at both the query-ranking and query-representation stages. First, the selection path calculates first-order local absolute differences from a subset of encoder channels and constructs a parameter-free structural saliency score. This score is used as complementary evidence to recalibrate the semantic Top-K ranking of RT-DETR, rather than replacing semantic confidence or redefining the query reference points. Second, after the Top-K indices have been determined, the enhancement path combines an image-level dynamic coefficient with the structural response at each selected location to perform residual multiplicative modulation of the corresponding query embedding. Thus, ASQS influences both which encoder locations are selected and how the selected queries are represented, without requiring an auxiliary saliency-prediction branch or additional saliency annotations. This two-stage use of local structural information constitutes the principal methodological distinction between ASQS and existing saliency-aware query designs.

In summary, current detection methods face three key limitations in tiny steel surface defect inspection. First, existing lightweight backbones prioritize computational compression but may fail to sufficiently preserve the fine-grained edge, texture, and directional information required for micro-defect recognition [23]. Second, conventional cross-scale fusion schemes rely on dense convolution stacks, introducing redundant computations while being insufficiently tailored to sparse and low-contrast steel defects [26]. Third, the semantics-only query initialization of vanilla RT-DETR can be suboptimal for weak-semantic defects with salient local structural variations [17].

Accordingly, the objective of this study is to improve the detection of weak-semantic and structurally distinctive steel surface defects while reducing the computational cost of the detector. To achieve this objective, this paper develops SA-DETR through defect-oriented lightweight reconstruction of the backbone and cross-scale feature fusion network, together with a structure-aware query initialization strategy. Specifically, O-EHGBlock is used to reduce redundant spatial computation while preserving fine-grained structural cues, StarC3 provides lightweight cross-scale feature interaction, and ASQS explicitly incorporates local structural information into both query ranking and query representation. These three modules address feature extraction, query initialization, and feature fusion, respectively, enabling SA-DETR to pursue a balanced trade-off among detection performance, localization quality, computational complexity, and inference efficiency.

## 3. Method

### 3.1. Overall Network Architecture

Tailored for lightweight and robust tiny steel surface defect detection, the proposed SA-DETR model comprises four core components: a lightweight feature extraction backbone, a hybrid encoding and cross-scale feature fusion neck, an Adaptive Structure-aware Query Selection (ASQS) module, and a Transformer-based detection decoder. The overall network architecture is visualized in Figure 1.

For an input steel surface image, HGStem first performs initial feature embedding and spatial downsampling. A stack of the proposed O-EHGBlock modules then executes hierarchical feature extraction to generate multi-scale feature representations across different network stages. Shallow features preserve high-resolution edge and texture details of tiny defects, while deep features with enlarged receptive fields capture high-level semantic information, jointly supporting comprehensive characterization of diverse steel surface defects.

All multi-stage backbone features are calibrated via 1 × 1 convolutions for consistent channel dimensions. The deepest semantic feature is processed by the Attention-based Intra-scale Feature Interaction (AIFI) module to capture long-range global context and enhance intra-scale feature discrimination. Subsequently, deep semantic features are bidirectionally fused with middle and shallow detail features. To reduce computational redundancy, the original RepC3 modules in RT-DETR-L are replaced with lightweight StarC3 modules, constructing an efficient fusion neck that achieves low-cost, high-quality cross-scale feature interaction and outputs three-scale fused feature maps.

Unlike vanilla RT-DETR that initializes queries purely based on semantic scores, SA-DETR incorporates the ASQS module at the encoder–decoder transition stage. During Top-K candidate selection, ASQS adaptively fuses semantic responses and local structural saliency cues of steel defects. A dynamic structural gating mechanism further enhances the structural representation of selected query tokens, effectively compensating for weak semantic responses of tiny, low-contrast defects. The optimized queries are finally fed into the Transformer decoder for defect classification and bounding box regression, realizing a complete end-to-end detection pipeline.

Overall, SA-DETR implements three targeted optimizations upon the RT-DETR-L baseline. The O-EHGBlock backbone balances lightweight computation and fine-grained structural feature preservation for tiny defects. The ASQS mechanism eliminates the query selection bias of semantics-only strategies for weak-semantic structural defects. The StarC3-based fusion neck reduces model complexity while maintaining sufficient cross-scale feature interaction. The synergistic design of the three modules achieves a favorable trade-off among detection accuracy, inference efficiency, and industrial deployment capability.

### 3.2. Lightweight Backbone Reconstruction Based on O-EHGBlock

Thin scratches, crazing and strip inclusions dominate tiny steel surface defects, which are characterized by ultra-small pixel coverage, low local contrast and distinct elongated directional textures. Conventional HGBlock relies on full-channel dense convolution for hierarchical feature aggregation. Stacking such modules introduces excessive spatial computation overhead; more critically, repeated multi-stage downsampling gradually erodes subtle edge and texture cues of micro-defects, severely degrading the model’s fine-grained perception capability. To resolve the trade-off between inference lightweightness and structural feature retention for elongated tiny defects, this work designs an Optimized Efficient Hierarchical Ghost Block (O-EHGBlock), whose structural layout is illustrated in Figure 2. The proposed module integrates multi-scale partial convolution (MS-PConv), a dedicated lightweight structural enhancement branch (LightBranch), and fused coordinate attention (FCA) deployed at deep network stages, forming a multi-path collaborative feature extraction paradigm.

MS-PConv performs spatial modeling on only part of the input channels. The remaining channels are preserved through an identity path. This reduces redundant spatial computation. LightBranch supplements the local edge and texture information that may be weakened by partial convolution. FCA is used only in the deep stages of the backbone. It models long-range positional information along the horizontal and vertical directions. This improves the perception of elongated defects and their directional structures.

A learnable scaling factor initialized to zero is also introduced into FCA. At the beginning of training, the attention branch therefore behaves approximately as an identity mapping. This reduces disturbance to the backbone feature distribution and improves training stability.

#### 3.2.1. Partial Spatial Modeling and Feature Preservation Based on MS-PConv

Let the input feature of O-EHGBlock be

(1) $$X\in R^{C\times H\times W}$$

where *C*, *H*, and *W* denote the number of channels, feature height, and feature width, respectively.

A conventional convolution performs spatial convolution on all input channels. To reduce unnecessary spatial computation, MS-PConv divides the input feature into a spatial convolution branch and an identity preservation branch according to a ratio *r*:

(2) $$X_{s},X_{i}=\mathrm{Split}\left(X,r\right),$$

where

(3) $$X_{s}\in R^{rC\times H\times W},\;X_{i}\in R^{(1-r)C\times H\times W}.$$

Here, $X_{s}$ denotes the features processed by spatial convolution, while $X_{i}$ denotes the features preserved through the identity path. In this study, *r* is set to 0.75 . Thus, only 75% of the channels are processed by spatial convolution. The remaining 25% retain their original features.

The output of the spatial branch is expressed as

(4) $$Y_{s}={\mathrm{Conv}}_{k\times k}\left(X_{s}\right)$$

The output of the convolution branch is then concatenated with the identity branch along the channel dimension. A 1×1 convolution is used for channel fusion:

(5) $$Y_{\mathrm{MS}}={\mathrm{Conv}}_{1\times 1}\left[\mathrm{Concat}(Y_{s},X_{i})\right]$$

This design avoids repeated spatial convolution on all input channels. It reduces computational cost while preserving part of the original features without spatial transformation. As a result, the excessive loss of small-defect information during deep feature extraction can be alleviated.

#### 3.2.2. Serial Hierarchical Feature Modeling and LightBranch-Based Structure Enhancement

O-EHGBlock retains the hierarchical feature aggregation strategy of HGBlock. Within the module, multiple MS-PConv layers are connected in series. This allows local features to be modeled progressively. The output of the *i*-th MS-PConv is defined as

(6) $$Z_{i}={\mathrm{MS}-\mathrm{PConv}}_{i}\left(Z_{i-1}\right),\;Z_{0}=X.$$

After *n* MS-PConv layers, the original input and the intermediate features from different levels are retained and concatenated:

(7) $$Y_{\mathrm{serial}}=\mathrm{Concat}\left(X,Z_{1},Z_{2},\ldots ,Z_{n}\right)$$

This hierarchical aggregation preserves local responses from different depths. It enables the module to obtain multilevel feature representations with relatively low computational cost.

However, applying spatial convolution to only part of the channels may lead to insufficient extraction of local edges and textures. To address this issue, a lightweight structure enhancement branch, termed LightBranch, is introduced. It directly performs a lightweight transformation on the original input.

LightBranch takes the original feature *X* as input. It consists of a 1×1 convolution and a 3×3 convolution. The branch is used to supplement local edge and texture responses:

(8) $$P_{\mathrm{struct}}=\mathrm{LightBranch}\left(X\right)$$

The serial hierarchical feature $Y_{\mathrm{serial}}$ is then concatenated with the structure-enhanced feature $P_{\mathrm{struct}}$ along the channel dimension:

(9) $$Y_{\mathrm{cat}}=\mathrm{Concat}\left(Y_{\mathrm{serial}},P_{\mathrm{struct}}\right)$$

Two successive 1×1 convolutions are then used for channel compression and output mapping. The final fused feature $Y_{f}$ is expressed as

(10) $$Y_{f}={\mathrm{Conv}}_{1\times 1}\left({\mathrm{Conv}}_{1\times 1}\left(Y_{\mathrm{cat}}\right)\right)$$

The primary branch realizes progressive multilevel feature aggregation, whereas LightBranch recovers and compensates the local structural cues inherent to the raw input feature. The collaborative design of these two branches empowers O-EHGBlock to cut redundant spatial computations without sacrificing discriminative representations of slender micro-defects.

#### 3.2.3. Fused Coordinate Attention

With network depth increasing, feature responses of micro steel defects are readily masked by intricate background textures and industrial noise. Typical surface flaws such as scratches and crazing exhibit elongated shapes distributed along horizontal, vertical or oblique orientations. Conventional channel attention and vanilla 2D global pooling fail to sufficiently capture directional positional cues of these strip-like defects. To address this limitation, we embed a Fused Coordinate Attention (FCA) module within deep O-EHGBlock stages, whose detailed architecture is illustrated in Figure 2c.

FCA first applies one-dimensional global pooling to the fused feature $Y_{f}$ along the horizontal and vertical directions:

(11) $$z^{h}\left(h\right)=\frac{1}{W}\sum_{i=1}^{W}Y_{f}\left(h,i\right)$$

(12) $$z^{w}\left(w\right)=\frac{1}{H}\sum_{j=1}^{H}Y_{f}\left(j,w\right)$$

where $z^{h}\left(h\right)$ and $z^{w}\left(w\right)$ preserve positional information along the horizontal and vertical directions, respectively. The context features from the two directions are then concatenated. A shared 1×1 convolution, batch normalization, and nonlinear activation are applied for feature transformation.

The transformed features are separated and mapped into horizontal and vertical attention weights:

(13) $$A_{h}=\sigma \left({\mathrm{Conv}}_{h}\left(z^{h}\right)\right)$$

(14) $$A_{w}=\sigma \left({\mathrm{Conv}}_{w}\left(z^{w}\right)\right)$$

where σ(·) denotes the Sigmoid activation function.

The attention-modulated feature is expressed as

(15) $$Y_{\mathrm{FCA}}=Y_{f}+\gamma \left(Y_{f}⊙A_{h}⊙A_{w}\right),$$

where ⊙ denotes element-wise multiplication and γ is a learnable scaling factor. In this work, γ is initialized to zero. Thus, FCA behaves approximately as an identity mapping at the beginning of training. This reduces disturbance from the attention branch to the backbone feature distribution and improves training stability.

When the input and output channels of the module are the same, O-EHGBlock further applies a residual connection:

(16) $$Y_{\mathrm{out}}=Y_{\mathrm{FCA}}+X$$

By combining directional information modeling, learnable scaling, and residual learning, FCA improves the sensitivity of deep features to defect locations and directional structures. It also maintains stable network training.

### 3.3. Adaptive Structure-Aware Query Selection

In RT-DETR, the quality of decoder query initialization dominates the downstream defect classification and bounding box regression performance. The vanilla RT-DETR conducts Top-K candidate screening purely based on semantic scores output by the encoder classification head, where feature locations bearing strong semantic responses are picked as initial object queries.

Such a semantic-driven selection paradigm achieves favorable performance for generic objects with distinct categorical semantics, yet it exhibits evident drawbacks when deployed for tiny steel surface defect detection. Micro flaws including scratches, crazing and inclusions only yield faint deep semantic responses, while their discriminative signatures mainly reside in conspicuous local edges, grayscale abrupt variations and geometric contours. If queries are screened merely by classification confidence, numerous defect regions with salient structural patterns but low semantic scores will be discarded from the Top-K candidate pool.

Furthermore, the discrete-ranking nature of the Top-K operation prevents continuous gradient-based optimization of structural feature cues. To mitigate the above bottlenecks, this work proposes an Adaptive Structure-Aware Query Selection (ASQS) mechanism.

Different from conventional attention or saliency-enhancement modules that mainly reweight feature responses, ASQS directly intervenes in the encoder-to-decoder query initialization process. Its key idea is not to replace semantic confidence with structural saliency, but to use local structural variation as complementary evidence for semantically ambiguous candidates. Specifically, ASQS consists of two complementary paths: a parameter-free selection path that recalibrates Top-K ranking using semantic and structural scores, and a differentiable enhancement path that adaptively modulates the representations of the selected queries. In this way, structural information contributes both to which locations are selected as queries and to how the selected queries are represented, which distinguishes ASQS from feature-level saliency enhancement alone.

ASQS consists of a selection path and an enhancement path, as shown in Figure 3 and Figure 4. The selection path uses structural saliency information to assist candidate query selection. The enhancement path further injects structural information into the selected query features through differentiable dynamic gating.

#### 3.3.1. Selection Path: Structure-Saliency-Assisted Query Selection Without Additional Learnable Parameters

After flattening the multiscale features produced by the hybrid encoder, the feature sequence is $F\in R^{B\times N\times C}$ , where *B* is the batch size, *N* is the total number of tokens obtained by flattening all feature scales, and *C* is the channel dimension.

Structural information mainly appears as local changes between neighboring spatial positions. Therefore, the flattened features are first reshaped into two-dimensional feature maps according to the spatial size of each scale:

(17) $$F_{s}\in R^{B\times C\times H_{s}\times W_{s}}$$

where $H_{s}$ and $W_{s}$ are the height and width of the feature map at the *s*-th scale, respectively.

To reduce the additional cost of structural saliency calculation, only part of the channels is used for local difference computation. The number of selected channels is defined as

(18) $$C_{\mathrm{sub}}=C/4$$

The corresponding subchannel feature is written as

(19) $${\hat{F}}_{s}=F_{s}\left[:,\;0:C/4,\;:,\;:\right]$$

The horizontal and vertical absolute differences are then calculated to measure the local structural changes between neighboring positions.

(20) $$\begin{array}{c} \left\{\begin{array}{c} G_{x}^{s}\left(b,c,i,j\right)=\left|{\hat{F}}_{s}\left(b,c,i,j+1\right)-{\hat{F}}_{s}\left(b,c,i,j\right)\right|\\ G_{y}^{s}\left(b,c,i,j\right)=\left|{\hat{F}}_{s}\left(b,c,i+1,j\right)-{\hat{F}}_{s}\left(b,c,i,j\right)\right| \end{array}\right. \end{array}$$

where $G_{x}^{s}$ and $G_{y}^{s}$ denote the horizontal and vertical feature-difference responses, respectively.

The adjacent difference operation reduces the spatial size by one along the corresponding direction. To keep the difference maps consistent with the original feature map, replication padding is applied according to the implementation. One column is added to the right side of the horizontal difference map, and one row is added to the bottom of the vertical difference map:

(21) $$\begin{array}{c} \left\{\begin{array}{c} {\tilde{G}}_{x}^{s}={\mathrm{Pad}}_{r}\left(G_{x}^{s}\right),\\ {\tilde{G}}_{y}^{s}={\mathrm{Pad}}_{b}\left(G_{y}^{s}\right) \end{array}\right. \end{array}$$

where ${\mathrm{Pad}}_{r}\left(\cdot \right)$ denotes replication padding with one column added to the right side of the feature map. Similarly, ${\mathrm{Pad}}_{b}\left(\cdot \right)$ adds one row to the bottom.

The structural responses in the two directions are then aggregated along the channel dimension. The structural saliency score at the *s*-th scale is calculated as

(22) $$S_{\mathrm{struct}}^{s}\left(b,i,j\right)=\frac{1}{C_{\mathrm{sub}}}\sum_{c=1}^{C_{\mathrm{sub}}}\left({\tilde{G}}_{x}^{s}\left(b,c,i,j\right)+{\tilde{G}}_{y}^{s}\left(b,c,i,j\right)\right)$$

To reduce the influence of response-magnitude differences among samples, Min–Max normalization is applied:

(23) $${\hat{S}}_{\mathrm{struct}}=\frac{S_{\mathrm{struct}}-\min\left(S_{\mathrm{struct}}\right)}{\max\left(S_{\mathrm{struct}}\right)-\min\left(S_{\mathrm{struct}}\right)+\varepsilon }$$

where ε is a small constant used to prevent division by zero. This operation maps the concatenated structural saliency scores of each sample to [0,1] . It also preserves the relative structural responses among different tokens in the same sample.

In the original RT-DETR, semantic scores are produced by the encoder classification head. Let the raw classification logits be

(24) $$P_{\mathrm{SEM}}=\mathrm{ClsHead}\left(F\right)$$

where $P_{\mathrm{SEM}}\in R^{B\times N\times M}$ , and *M* is the number of defect categories. According to the implementation, neither Sigmoid nor Softmax is applied to the classification logits during query selection. Instead, the maximum value along the category dimension is used directly. Thus, the semantic response score of the *n*-th token is

(25) $$S_{\mathrm{sem}}=\max_{m\in \{1,\ldots ,M\}}P_{\mathrm{sem}}\left(b,n,m\right).$$

The semantic score and the structural saliency score are then combined to obtain the final query selection score:

(26) $$S_{\mathrm{fused}}=S_{\mathrm{sem}}+\beta {\hat{S}}_{\mathrm{struct}},$$

where β controls the contribution of structural saliency during query selection. In this study, β is set to 0.3 .

It should be noted that $S_{\mathrm{sem}}$ contains raw classification logits without probability activation, whereas ${\hat{S}}_{\mathrm{struct}}$ is normalized to [0,1] . Min–Max normalization is not intended to make the two terms share the same numerical range. Instead, it limits the variation of the structural term. Under the control of β , the structural score acts as a stable additive bias and avoids dominating the semantic ranking.

Finally, Top-*K* query selection is performed according to the fused score:

(27) $$J=\mathrm{TopK}\left(S_{\mathrm{fused}},K\right)$$

where J denotes the index set of the selected queries, and *K* is the number of queries.

Unlike the original RT-DETR, which ranks queries using only classification logits, the proposed selection path uses local structural saliency as auxiliary information. Regions with relatively weak semantic responses but clear local edges or structural changes can therefore receive higher query priorities. This improves the coverage of candidate regions for small defects.

The structural saliency calculation only involves channel slicing, local difference operations, replication padding, and channel aggregation. It introduces no additional learnable parameters. Moreover, the structural score is used only as a weighted auxiliary term rather than an independent query selection criterion.

#### 3.3.2. Enhancement Path: Differentiable Dynamic Structural Gating

The selection path adjusts the query scores using structural saliency. It gives higher priority to regions with clear structural changes. However, Top-*K* index selection is still a discrete ranking operation. Gradients cannot directly optimize which positions are selected through the indices. To make better use of structural information, a differentiable enhancement path is introduced after the query indices are determined. This path adaptively modulates the selected query embeddings. Structural information can therefore participate in the subsequent decoding process through query representation learning.

First, global average pooling is applied to the encoder feature *F* along the token dimension. This produces an image-level global context representation:

(28) $$F_{g}=\frac{1}{N}\sum_{n=1}^{N}F\left(:,n,:\right)$$

where $F_{g}\in R^{B\times C}$ .

The global context feature is then fed into a lightweight multilayer perceptron. A Sigmoid activation function is used to generate an image-level dynamic structural modulation coefficient:

(29) $$\alpha_{\mathrm{dyn}}=\sigma \left(\mathrm{MLP}(F_{g})\right)$$

where σ(·) denotes the Sigmoid activation function. The coefficient $\alpha_{\mathrm{dyn}}$ controls the overall strength of structural enhancement for each input image according to its global context.

Based on the Top-*K* index set I obtained from the selection path, the initial query embeddings are gathered from the encoder features.

(30) $$Q_{\mathrm{raw}}=\mathrm{Gather}\left(F,J\right)$$

where $Q_{\mathrm{raw}}\in R^{B\times K\times C}$ .

The structural saliency responses at the same positions are extracted from the normalized structural scores:

(31) $$S_{\mathrm{topk}}=\mathrm{Gather}\left({\hat{S}}_{\mathrm{struct}},J\right)$$

where $S_{\mathrm{topk}}\in R^{B\times K\times 1}$ .

The term $S_{\mathrm{topk}}$ describes the local structural saliency of each selected query position. In contrast, $\alpha_{\mathrm{dyn}}$ controls the overall enhancement strength according to the image-level context. Together, they form a query-level dynamic structural modulation factor:

(32) $$M_{\mathrm{struct}}=\alpha_{\mathrm{dyn}}\cdot S_{\mathrm{topk}}$$

The initial query embeddings are then enhanced as

(33) $$Q_{\mathrm{final}}=Q_{\mathrm{raw}}⊙\left(1+M_{\mathrm{struct}}\right)$$

where ⊙ denotes element-wise multiplication. The modulation factor $M_{\mathrm{struct}}$ is broadcast along the channel dimension and therefore acts on all channels of each query embedding.

Both $\alpha_{\mathrm{dyn}}$ and $S_{\mathrm{topk}}$ are constrained by Sigmoid activation or Min–Max normalization. Thus, the modulation works as a residual multiplicative enhancement. When the structural response is weak, the query feature remains nearly unchanged. When the structural response is strong, the corresponding query embedding is enhanced.

This enhancement path does not change the discrete nature of Top-*K* index selection. Instead, it applies differentiable dynamic modulation after the query indices are determined. Gradients can therefore propagate to the selected encoder features and the branch that generates $\alpha_{\mathrm{dyn}}$ . In this way, structural information is used not only to select candidate positions but also to improve the representation of the selected queries.

By combining the selection path and the enhancement path, ASQS improves query initialization at two levels. The selection path supplements semantic scores with structural saliency. The enhancement path adjusts the selected query representations according to the image context and local structural responses. This joint design improves the representation of small defects and defects with elongated structures.

### 3.4. Lightweight Feature Fusion Based on StarC3

The cross-scale feature fusion network in the original RT-DETR-L uses RepC3 to exchange information among features at different scales. RepC3 provides strong feature transformation capability. However, it is repeatedly used in multiple scale branches. This increases the number of parameters and computational cost, which makes lightweight deployment on resource-constrained devices more difficult.

To reduce the computational cost of the neck, a lightweight feature fusion module named StarC3 is designed to replace RepC3. Its structure is shown in Figure 5.

StarC3 retains the dual-branch feature flow of the C3 structure. The main branch contains StarBlocks, which perform multiplicative nonlinear interaction through element-wise multiplication between two feature branches. The other branch uses a shorter feature path to preserve part of the original information. A LayerScale mechanism is also introduced into StarBlock to control the output strength of the multiplicative interaction branch and improve training stability.

#### 3.4.1. StarBlock

StarBlock is the core computational unit of StarC3. Given an input feature $X\in R^{B\times C\times H\times W}$ , *B* is the batch size, *C* is the number of channels, and *H* and *W* are the height and width of the feature map, respectively.

StarBlock first uses two independent 1×1 convolution branches to map the input feature into two different feature subspaces:

(34) $$F_{i}=\phi \left({\mathrm{Conv}}_{1\times 1}^{\left(i\right)}\left(X\right)\right),i=1,2$$

where ϕ(·) denotes the normalization and nonlinear activation operations in the convolution module.

The two features are then combined through element-wise multiplication:

(35) $$F_{\mathrm{star}}=F_{1}⊙F_{2}$$

where ⊙ denotes element-wise multiplication.

The Star Operation introduces multiplicative nonlinear interaction between the two feature branches at the same spatial position. This strengthens the relationship between different feature mappings. Since no additional large-kernel spatial convolution is required, the feature interaction can be improved with relatively low computational cost.

To reduce the disturbance caused by the multiplicative branch at the beginning of training, we introduce a channel-wise learnable LayerScale parameter $\gamma \in R^{C}$ .

The output of StarBlock is expressed as

(36) $$Y=X+\gamma ⊙F_{\mathrm{star}}$$

In this study, γ is initialized to a small value of 0.01 . Therefore, StarBlock behaves approximately as an identity mapping at the beginning of training:

(37) Y≈X

During training, γ is updated through backpropagation. It gradually adjusts the contribution of the multiplicative interaction branch to the output feature. This design improves the stability of the training process.

#### 3.4.2. StarC3 Module

Based on StarBlock, a StarC3 module is constructed to replace RepC3 in the original CCFM. StarC3 retains the dual-branch feature flow of the C3 structure. One branch uses stacked StarBlocks for nonlinear feature interaction, while the other branch follows a short path to preserve part of the original information.

Specifically, the input feature *X* is first processed by two independent 1×1 convolution branches:

(38) $$Y_{1}={\mathrm{StarBlock}}^{n}\left({\mathrm{Conv}}_{1\times 1}^{\left(1\right)}\left(X\right)\right),$$

(39) $$Y_{2}={\mathrm{Conv}}_{1\times 1}^{\left(2\right)}\left(X\right)$$

where *n* denotes the number of stacked StarBlocks. The outputs of the two branches are then concatenated along the channel dimension:

(40) $$Y_{\mathrm{cat}}=\mathrm{Concat}\left(Y_{1},Y_{2}\right)$$

A 1×1 convolution is applied to fuse the concatenated features:

(41) $$Y_{\mathrm{out}}={\mathrm{Conv}}_{1\times 1}\left(Y_{\mathrm{cat}}\right)$$

When the input and output features have the same number of channels, a residual connection is further introduced:

(42) $$Y_{\mathrm{final}}=X+Y_{\mathrm{out}}$$

The dual-branch structure preserves part of the input features through the short path. Meanwhile, the StarBlock branch provides lightweight nonlinear interaction. This design reduces feature loss during successive transformations and lowers the computational cost caused by dense spatial convolutions.

From the perspective of computational complexity, the cost of a standard 3×3 convolution can be approximated as $O\left(9C^{2}HW\right)$ , whereas the cost of a 1×1 convolution is $O\left(C^{2}HW\right)$ . Compared with feature fusion modules that rely heavily on 3×3 convolutions, StarC3 uses fewer dense spatial convolutions. It instead combines lightweight channel mapping with element-wise multiplication for feature interaction. This helps reduce the computational cost of the neck.

In addition, LayerScale controls the contribution of the multiplicative branch at the beginning of training. This makes StarC3 suitable for multiscale feature fusion in an end-to-end detection framework.

In summary, StarC3 combines a dual-branch feature flow, lightweight channel mapping, multiplicative interaction through the Star Operation, and stable modulation through LayerScale. It reduces model complexity while maintaining effective cross-scale feature interaction. Therefore, it supports the lightweight design of SA-DETR.

## 4. Experiments and Analysis

### 4.1. Experimental Environment and Training Settings

The effectiveness of SA-DETR is evaluated on the NEU-DET [28] steel surface defect dataset and the PCB defect dataset [29]. NEU-DET contains 1800 images from six defect categories: crazing, inclusion, patches, pitted surface, rolled-in scale, and scratches. The PCB dataset contains 693 images from six defect categories: missing hole, mouse bite, open circuit, short, spur, and spurious copper. This dataset is used to evaluate the applicability of the model to different industrial defect scenarios.

Both datasets are divided into training, validation, and test sets at a ratio of 8:1:1. For the PCB dataset, the training, validation, and test sets contain 560, 70, and 63 images, respectively. All input images are resized to 640×640 pixels. Both datasets are divided into training, validation, and test sets for model development and evaluation.

Considering the relatively small size of the PCB dataset, five independent stratified random splits were performed to evaluate the robustness of the results. For each split, the training, validation, and test sets were generated using the same partitioning protocol but different random seeds while preserving the class distribution as consistently as possible. RT-DETR-L and SA-DETR were independently trained on each split under identical training settings. The mean and standard deviation over the five runs are reported. All experiments are implemented using PyTorch 2.1 and conducted on an NVIDIA RTX 4090 GPU. AdamW is used as the optimizer. The initial learning rate and weight decay are set to $1\times 10^{-4}$ and $1\times 10^{-3}$ , respectively. The batch size is set to 4, and the maximum number of training epochs is 500.

An early stopping strategy is applied during training. Training is terminated when the validation performance does not improve for 50 consecutive epochs. All models are trained from scratch without pretrained weights. Except for differences in network architecture, all comparison models use the same dataset splits and basic training settings.

### 4.2. Evaluation Metrics

To evaluate the detection performance comprehensively, Precision (*P*), Recall (*R*), mAP@0.5 ( ${\mathrm{mAP}}_{50}$ ), and mAP@0.5:0.95 ( ${\mathrm{mAP}}_{50:95}$ ) [30] are used as accuracy metrics. The number of parameters (Params), floating-point operations (GFLOPs), and inference speed (FPS) are also reported to evaluate model complexity and deployment efficiency.

Precision and Recall are defined as

(43) $$P=\frac{\mathrm{TP}}{\mathrm{TP}+\mathrm{FP}}$$

(44) $$R=\frac{\mathrm{TP}}{\mathrm{TP}+\mathrm{FN}}$$

where TP , FP , and FN denote true positives, false positives, and false negatives, respectively.

Average Precision (AP) is defined as the area under the Precision–Recall curve:

(45) $$\mathrm{AP}=\int_{0}^{1}P\left(R\right)\;dR$$

The mean Average Precision (mAP) is the average AP over all defect categories:

(46) $$\mathrm{mAP}=\frac{1}{M}\sum_{i=1}^{M}{\mathrm{AP}}_{i}$$

where *M* denotes the number of defect categories.

In this study, ${\mathrm{mAP}}_{50}$ is calculated at an Intersection over Union (IoU) threshold of 0.5. The ${\mathrm{mAP}}_{50:95}$ is averaged over IoU thresholds from 0.50 to 0.95 with a step size of 0.05.

Params and GFLOPs are used to measure model complexity, while Frames Per Second (FPS) is used to measure inference speed. Higher Precision, Recall, mAP, and FPS values indicate better performance. Lower Params and GFLOPs values indicate a lighter model that is more suitable for deployment on industrial edge devices. To further characterize the accuracy–efficiency trade-off, ${\mathrm{mAP}}_{50}$ /GFLOP and ${\mathrm{mAP}}_{50:95}$ /GFLOP are introduced as supplementary efficiency indicators, defined as the ratio of the corresponding mAP value to GFLOPs. Higher values indicate higher detection performance per unit of computational cost.

### 4.3. Comparison Experiments on the NEU-DET Dataset

To evaluate the overall performance of SA-DETR in the detection of steel surface defects, it is compared to Faster R-CNN [3], RT-DETR-ResNet50, RT-DETR-ResNet101, YOLOv10-L [31], YOLOv12-X [32] and RT-DETR-L. The experimental results are presented in Table 1.

As shown in Table 1, SA-DETR achieves a favorable balance between detection accuracy and model complexity. Compared with the RT-DETR-L baseline, its Precision and Recall increase from 68.2% and 66.9% to 73.3% and 69.7%, respectively. Its ${\mathrm{mAP}}_{50}$ increases from 69.4% to 73.9%, an improvement of 4.5 percentage points. The ${\mathrm{mAP}}_{50:95}$ increases from 40.7% to 42.7%, an improvement of 2.0 percentage points.

At the same time, the number of parameters decreases from 32.8 M to 21.0 M, while GFLOPs decrease from 108.0 to 57.8. These values correspond to reductions of approximately 36.0% and 46.5%, respectively. The inference speed also increases from 80.7 FPS to 103.0 FPS. These results show that SA-DETR improves detection accuracy while effectively reducing model complexity.

The smaller improvement in ${\mathrm{mAP}}_{50:95}$ indicates that the gain of SA-DETR is more pronounced at moderate IoU thresholds than under stricter localization criteria. This is mainly because the proposed modules focus on feature representation, query initialization, and lightweight feature fusion rather than directly optimizing bounding-box regression. Moreover, the ambiguous boundaries of thin and irregular defects make high-IoU evaluation more sensitive to small localization errors. Nevertheless, the increase from 40.7% to 42.7% demonstrates that SA-DETR still improves localization quality, while further gains may require dedicated regression or boundary-refinement strategies.

YOLOv12-X achieves a higher ${\mathrm{mAP}}_{50}$ of 78.5%, compared with 73.9% for SA-DETR. However, SA-DETR uses only 21.0 M parameters and 57.8 GFLOPs, compared with 59.1 M and 199.9 GFLOPs for YOLOv12-X, while increasing the inference speed from 49.0 FPS to 103.0 FPS. SA-DETR also achieves a higher ${\mathrm{mAP}}_{50:95}$ of 42.7% than the 41.4% of YOLOv12-X. Therefore, the contribution of SA-DETR lies primarily in its accuracy–efficiency trade-off rather than maximizing a single accuracy metric.

To further quantify this trade-off, ${\mathrm{mAP}}_{50}$ /GFLOP and ${\mathrm{mAP}}_{50:95}$ /GFLOP are introduced as supplementary efficiency indicators. SA-DETR achieves values of 1.28 and 0.74, respectively, compared with 0.39 and 0.21 for YOLOv12-X and 0.64 and 0.38 for RT-DETR-L, demonstrating higher detection performance per unit of computational cost.

### 4.4. Validation on the PCB Dataset

To further evaluate the applicability of the proposed method to different industrial defect scenarios, RT-DETR-L and SA-DETR are retrained and tested on the PCB defect dataset. Considering the relatively small size of the PCB dataset, five independent stratified random splits are used to reduce the influence of a specific data partition. Both models are independently trained and evaluated on each split, and the results are reported as mean ± standard deviation. The results are presented in Table 2.

Across the five data splits, SA-DETR achieves an average Precision of 91.1% ± 2.4%, compared with 87.0% ± 1.1% for RT-DETR-L. The average Recall also increases slightly from 83.4% ± 2.0% to 83.8% ± 1.7%. Meanwhile, ${\mathrm{mAP}}_{50}$ improves from 85.7% ± 0.9% to 87.4% ± 1.2%, and ${\mathrm{mAP}}_{50:95}$ increases from 42.8% ± 1.3% to 44.4% ± 0.6%. These results show that the performance improvements are maintained across different data partitions rather than depending on a single split.

Notably, the decrease in Recall observed in the original single-split experiment is not maintained across the repeated evaluations. The mean Recall of SA-DETR is slightly higher than that of RT-DETR-L over the five splits, suggesting that the previous decrease may have been influenced by the limited test-set size and the specific data partition. This observation further highlights the importance of repeated evaluation when assessing model performance on small datasets.

Nevertheless, because the PCB dataset remains relatively small and differs substantially from steel surface images in texture and structural characteristics, these results should be interpreted as preliminary evidence of the applicability of SA-DETR to another industrial defect scenario rather than conclusive evidence of broad cross-domain generalization. Further validation on larger and more diverse industrial datasets is required to assess its generalization capability more comprehensively.

### 4.5. Ablation Experiments

To further analyze the contribution of each proposed module, RT-DETR-L is used as the baseline. O-EHGBlock, ASQS, StarC3, and their different combinations are introduced under the same training settings. The ablation results are presented in Table 3.

As shown in Table 3, introducing O-EHGBlock alone increases ${\mathrm{mAP}}_{50}$ from 69.4% to 73.5%. Meanwhile, the number of parameters and GFLOPs decrease to 27.3 M and 89.2, respectively. These results show that O-EHGBlock reduces backbone complexity while improving the representation of small defects. However, ${\mathrm{mAP}}_{50:95}$ decreases slightly from 40.7% to 40.5%. This suggests that O-EHGBlock mainly improves defect representation and coverage, while its benefit for precise localization at high IoU thresholds is limited.

When only ASQS is introduced, ${\mathrm{mAP}}_{50}$ and ${\mathrm{mAP}}_{50:95}$ increase to 72.4% and 41.3%, respectively. The parameter count and GFLOPs remain almost unchanged. This confirms that structure-saliency-assisted query initialization improves detection performance with little additional computational cost.

After replacing RepC3 with StarC3, the parameter count and GFLOPs decrease to 26.5 M and 76.5, respectively. At the same time, ${\mathrm{mAP}}_{50}$ increases to 71.5%. This indicates that StarC3 can reduce the computational cost of cross-scale feature fusion while maintaining effective feature interaction.

The module combinations further reveal accuracy–efficiency trade-offs. O-EHGBlock + ASQS achieves the highest Precision of 75.5%, whereas the complete SA-DETR achieves higher Recall (69.7%), ${\mathrm{mAP}}_{50}$ (73.9%), and ${\mathrm{mAP}}_{50:95}$ (42.7%), while reducing Params from 27.3 M to 21.0 M and GFLOPs from 89.2 to 57.8. Similarly, O-EHGBlock + StarC3 reaches the highest inference speed of 107.5 FPS but provides lower detection accuracy than the complete model. These results show that maximizing a single metric does not necessarily provide the best overall performance. Therefore, the final architecture is selected to balance detection accuracy, localization quality, computational complexity, and inference efficiency.

### 4.6. Visualization of Detection Results

As shown in Figure 6, with the confidence threshold set to 0.6, SA-DETR demonstrates robust detection performance across the six defect categories. Compared with RT-DETR-L, SA-DETR reduces missed detections for crazing defects and provides more reliable predictions for patches, inclusions, and scratches. The two models show comparable performance for rolled-in scale defects. These qualitative results further demonstrate the effectiveness of SA-DETR in detecting weak-texture and low-contrast surface defects.

### 4.7. Parameter Sensitivity Analysis

To examine whether the selected hyperparameters were reasonable, sensitivity experiments were conducted for the channel ratio $r_{c}$ and the structural weighting coefficient β , while all other training settings were kept unchanged. Here, $r_{c}$ denotes the proportion of channels processed by the spatial feature extraction operation in O-EHGBlock, whereas the remaining channels retain a lightweight information propagation path. The coefficient β controls the contribution of local structural information to the semantic query-ranking score in ASQS.

As shown in Table 4, increasing $r_{c}$ from 0.500 to 0.750 improves Precision from 68.5% to 73.3% and mAP_50_ from 72.5% to 73.9%, while ${\mathrm{mAP}}_{50:95}$ increases slightly from 42.5% to 42.7%. When $r_{c}$ is further increased to 0.875 and 1.000, both detection accuracy and computational efficiency deteriorate. In particular, mAP_50_ decreases to 71.7% and 70.5%, respectively, while GFLOPs increase from 57.8 to 62.2 and 67.2. These results indicate that applying spatial operations to all channels does not necessarily improve defect representation and instead introduces additional computation. Although $r_{c}=0.500$ provides a more computationally efficient configuration and achieves a slightly higher Recall, $r_{c}=0.750$ obtains the highest mAP_50_ and mAP_50:95_ together with substantially lower computational complexity than the original RT-DETR-L. Therefore, $r_{c}=0.750$ was selected as an accuracy-oriented balance between structural feature extraction and computational efficiency. The $r_{c}=0.500$ configuration may serve as an alternative for devices with stricter computational constraints.

The results for β show that an intermediate contribution of structural information is beneficial to query initialization. When β is set to 0.1 or 0.2, the structural cues have a limited influence on semantic query ranking, resulting in mAP_50_ values of 70.2% and 70.6%, respectively. Increasing β to 0.3 produces the highest mAP_50_ of 73.9% and mAP_50:95_ of 42.7%. Although β=0.4 yields the highest Precision of 75.6%, its Recall decreases to 66.3%, indicating that excessive emphasis on structural responses may suppress semantically informative candidates and produce an unfavorable precision–recall trade-off. When β=0.5 , the performance partially recovers but remains below that obtained with β=0.3 . Therefore, β=0.3 was adopted because it provides the most favorable overall detection performance rather than maximizing a single metric.

Overall, the sensitivity results demonstrate that the selected parameters are supported by empirical comparisons. The model maintains competitive performance within moderate parameter settings, whereas excessively large channel-processing ratios or structural weights lead to accuracy degradation.

### 4.8. Sustainability Discussion

To discuss the potential sustainability benefits of the proposed method for green manufacturing, we consider model computational complexity as an indirect indicator of deployment resource demand. GFLOPs reflects the amount of floating-point computation required by a model rather than directly determining the instantaneous power consumption of the hardware. Under comparable hardware and software conditions, reducing computational workload generally has the potential to decrease execution time and energy consumption; however, actual power usage is also affected by factors such as memory access, hardware utilization, operator implementation, and the underlying computing platform. Therefore, the 46.5% reduction in GFLOPs achieved by SA-DETR should be interpreted as evidence of substantially lower computational demand and potential energy-saving capability, rather than as a directly measured reduction in electrical energy consumption.

In practical steel inspection scenarios, where visual inspection systems may operate continuously for extended periods, lower computational demand can reduce the hardware resources required for long-term operation and may contribute to lower energy consumption. In addition, improved defect detection can reduce the risk of defective products entering subsequent processing stages, thereby potentially avoiding unnecessary material consumption, rework, and secondary processing. These effects may contribute to reducing the use of raw materials, water, and energy associated with defective production. Nevertheless, direct measurements of device-level energy consumption, electricity usage, and carbon emissions are beyond the scope of the present study. Therefore, the sustainability advantage of SA-DETR is primarily reflected in its reduced computational requirements and its potential to support more resource-efficient industrial quality inspection.

## 5. Conclusions

This paper proposes SA-DETR, a lightweight structure-aware detector based on RT-DETR-L to tackle three critical bottlenecks of tiny steel defect detection: lost fine-grained structural features, semantic-biased query selection, and heavy cross-scale fusion computation. Three dedicated modules are designed for targeted optimization. The O-EHGBlock backbone adopts partial convolution, a structural compensation branch, and fused coordinate attention to cut redundant calculations while preserving edge and directional cues of slender micro-defects. The ASQS mechanism fuses semantic scores and structural saliency for Top-K selection, with differentiable gating to enhance weak-semantic defect queries without extra learnable parameters. The StarC3 fusion module replaces cumbersome RepC3 via lightweight multiplicative interaction and LayerScale, drastically lowering neck complexity. Validated on NEU-DET and PCB datasets, SA-DETR outperforms RT-DETR-L by 4.5 percentage points in mAP@0.5, reducing parameters by 36.0% and GFLOPs by 46.5%, while lifting inference speed to 103.0 FPS. Comparative and ablation experiments verify the complementary effectiveness of all three modules, delivering a favorable accuracy–efficiency trade-off for industrial edge deployment.

## Figures and Tables

**Figure 1:**
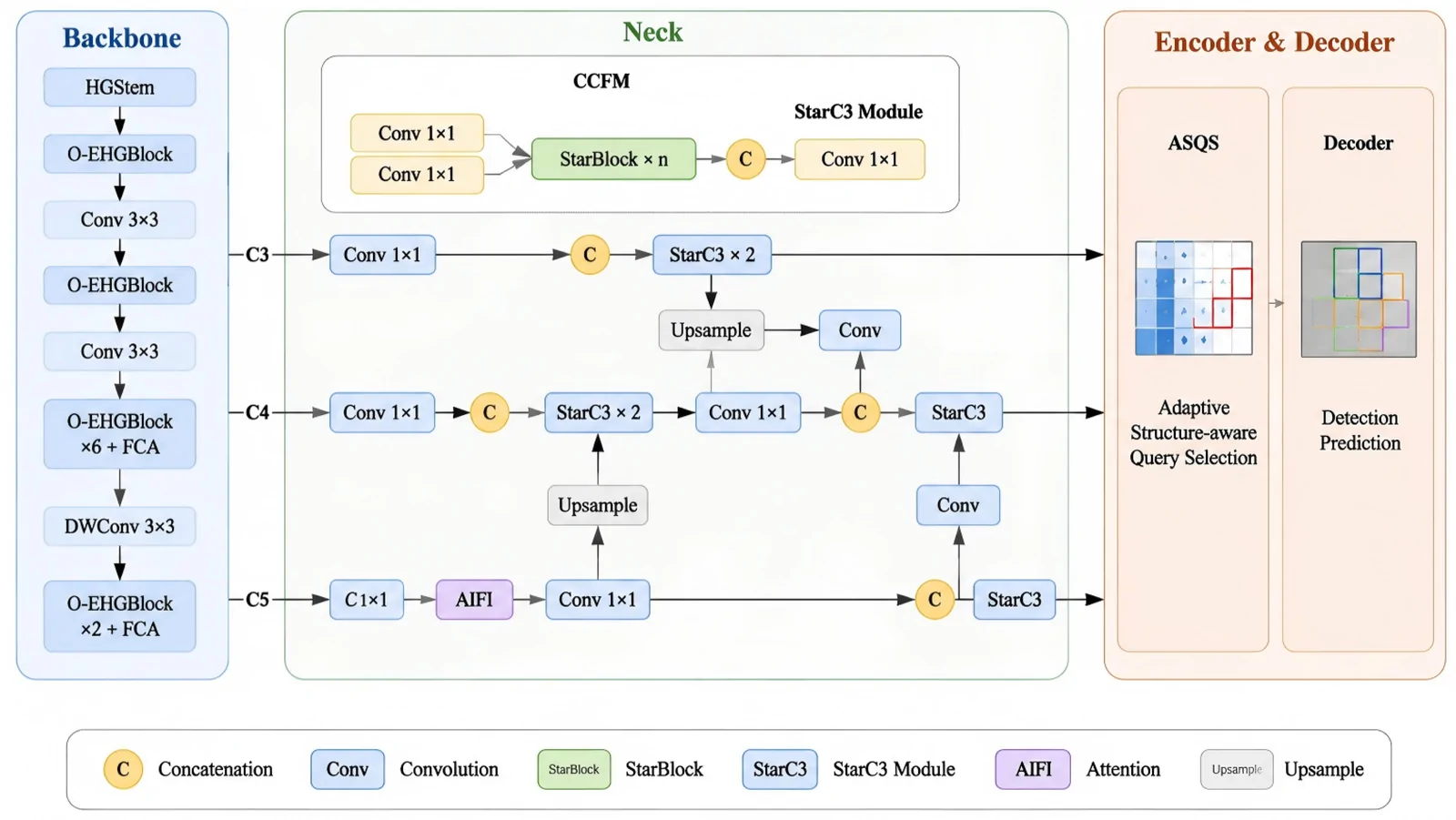
Overall network architecture of SA-DETR.

**Figure 2:**
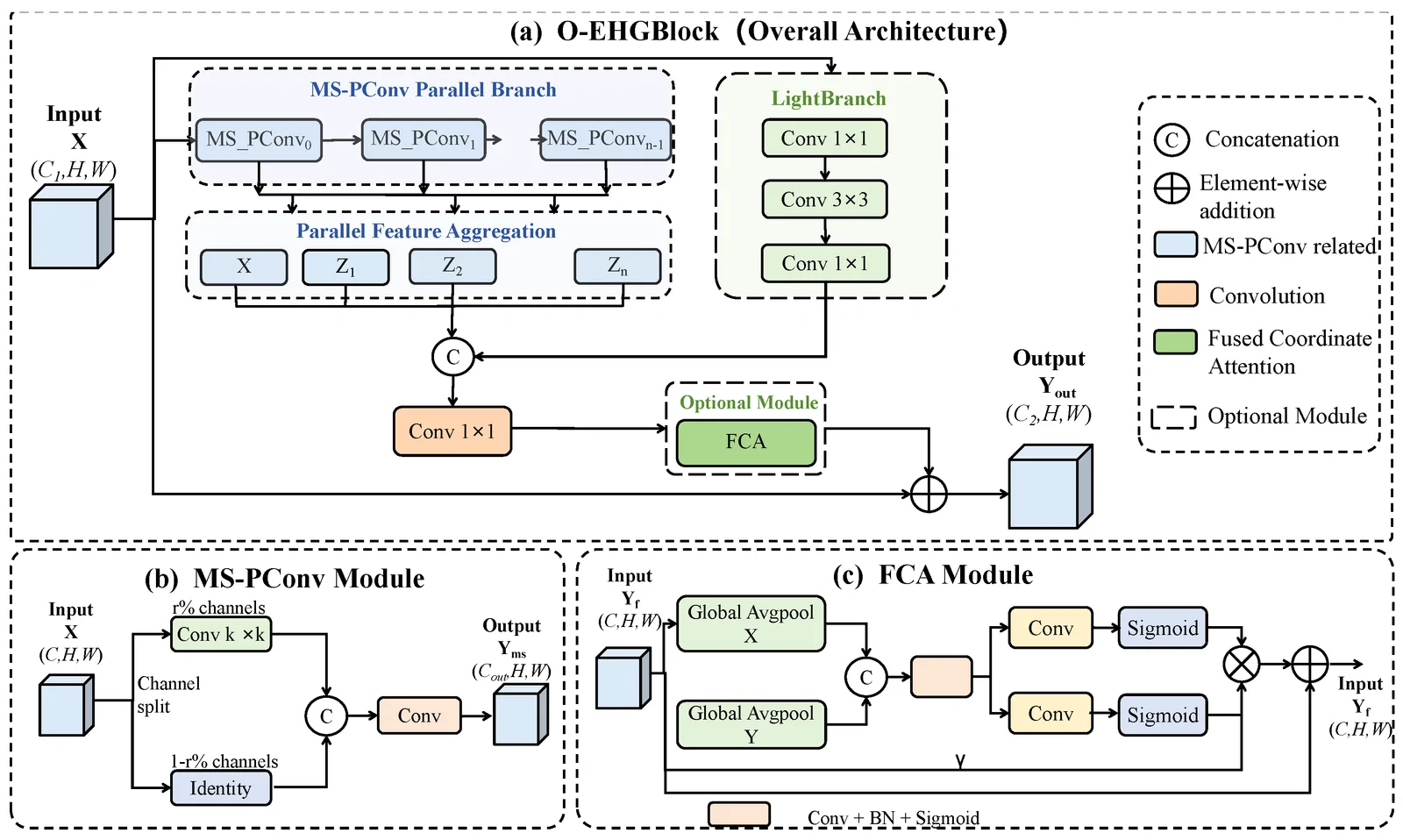
The network architecture of OEHGBlock.

**Figure 3:**
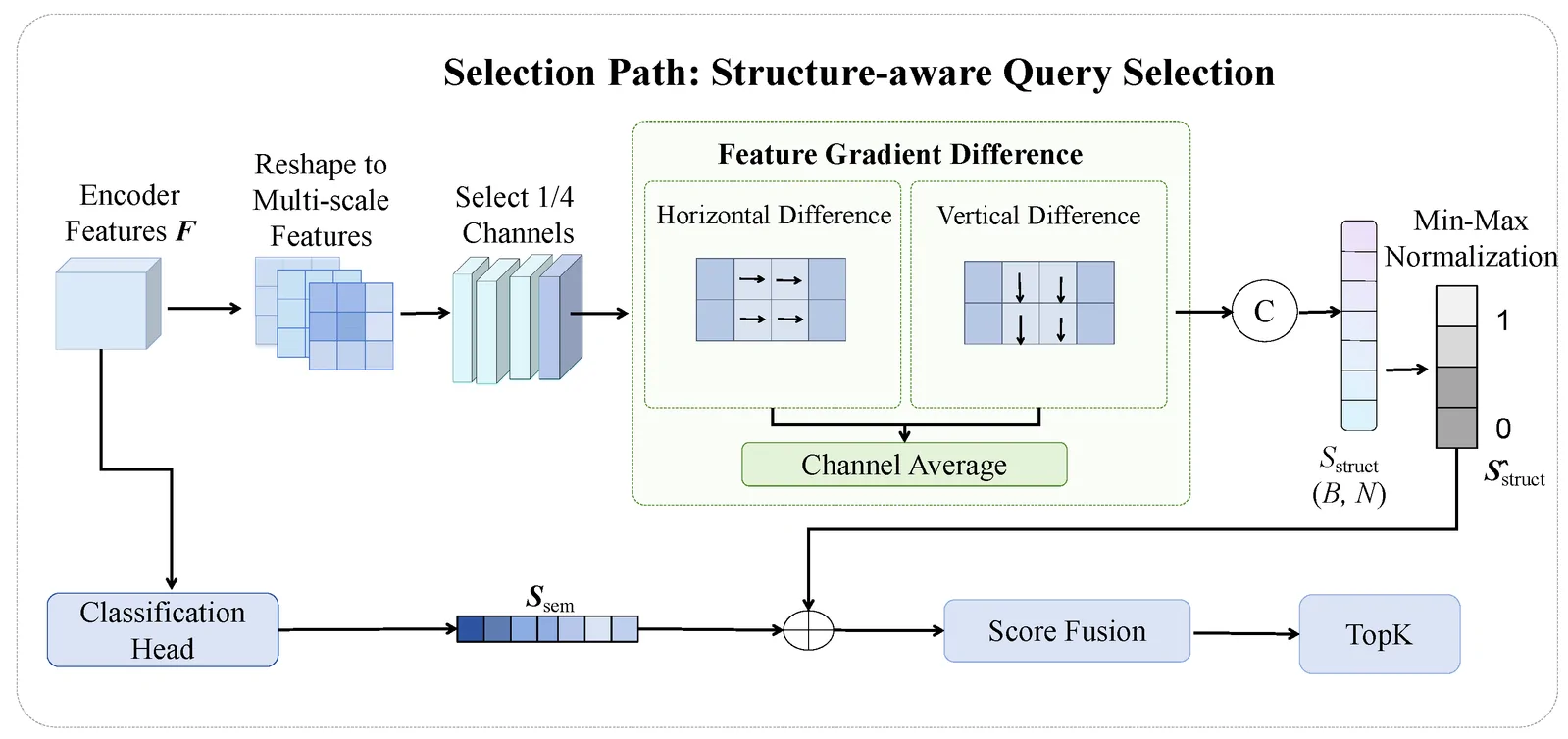
Architecture of the selection path in the proposed ASQS mechanism.

**Figure 4:**
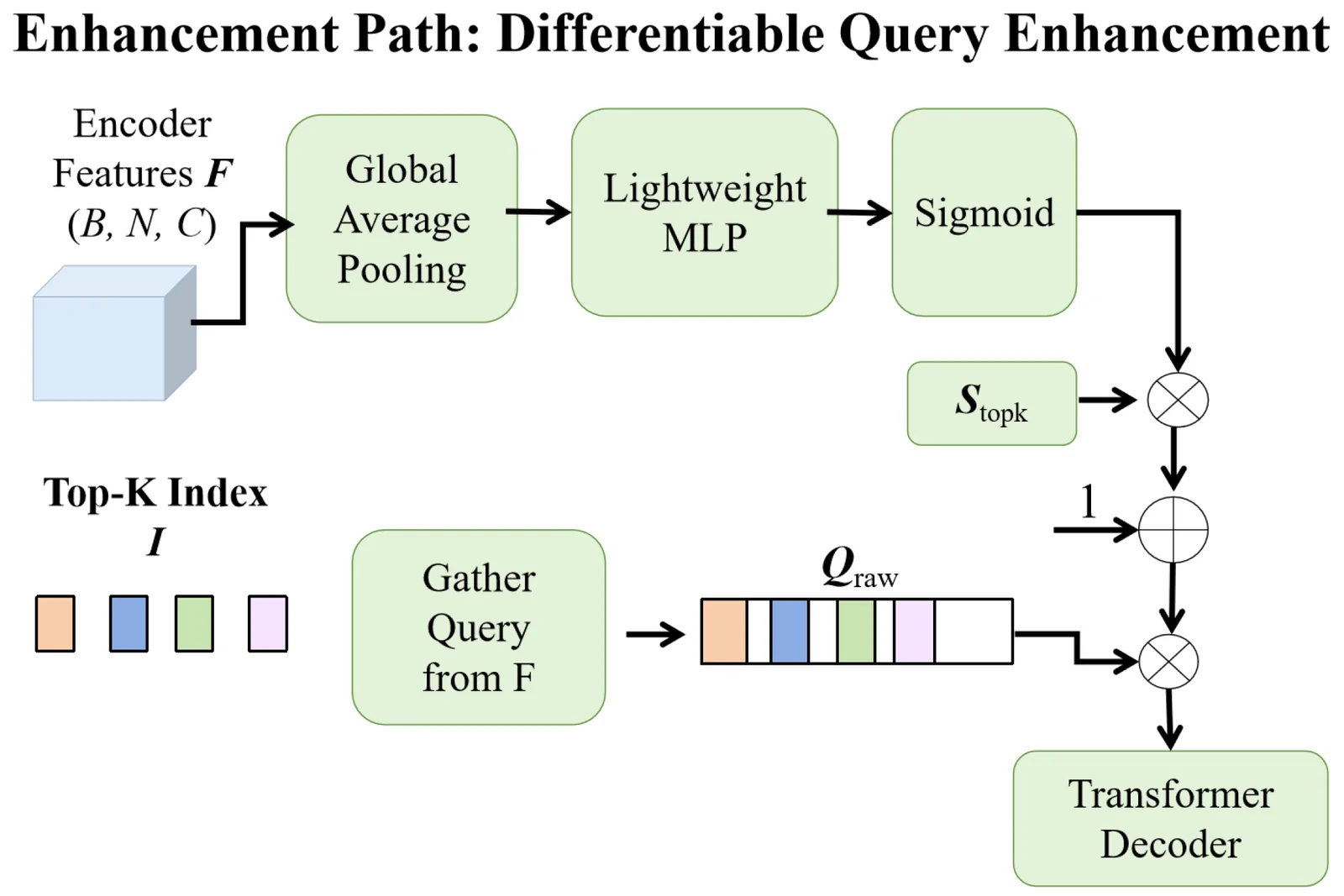
Architecture of the enhancement path in the proposed ASQS mechanism.

**Figure 5:**
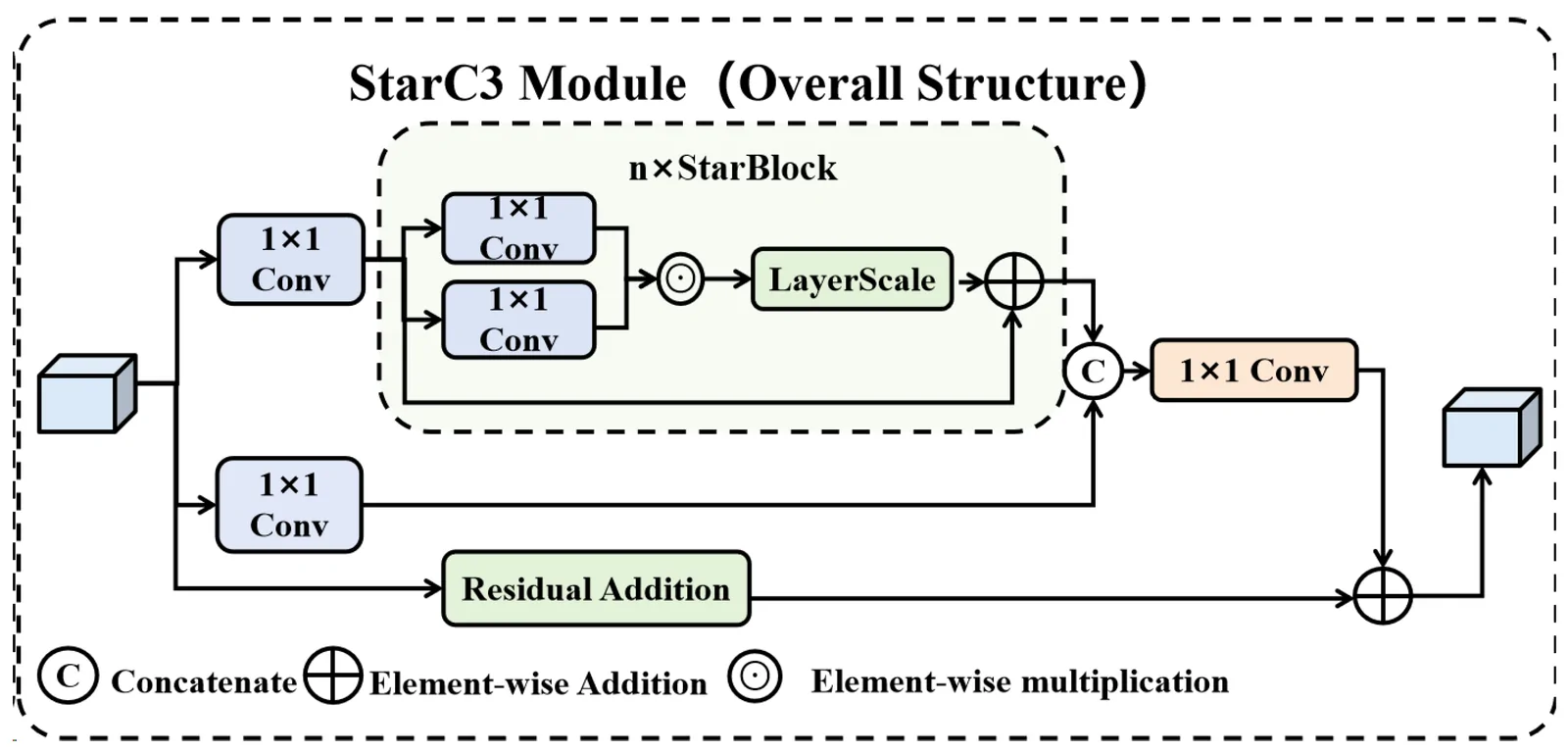
Architecture of the proposed StarC3 module.

**Figure 6:**
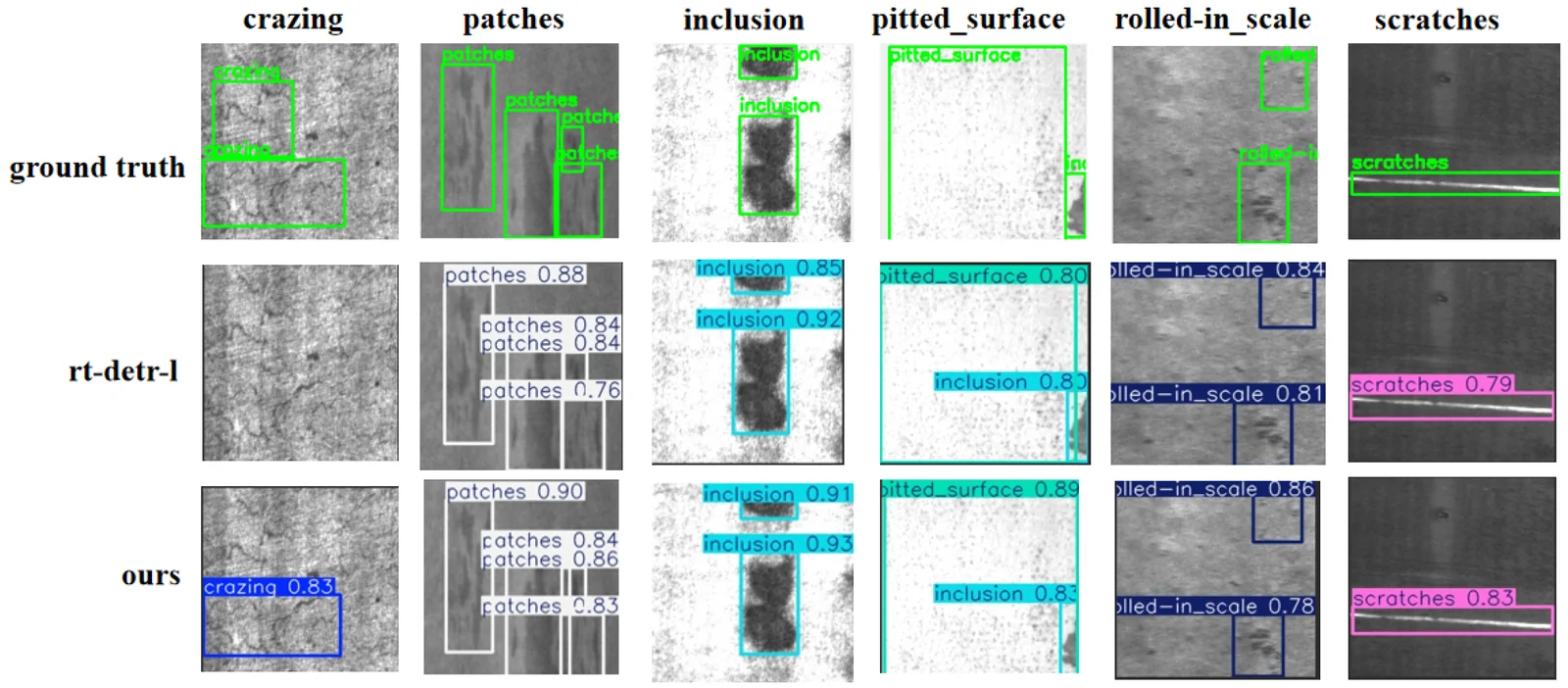
Visualization comparison of detection results between RT-DETR-L and SA-DETR on the NEU-DET dataset.

**Table 1:** Performance comparison of different object detection models on the NEU-DET dataset.

Model	P (%)	R (%)	${\mathrm{mAP}}_{50}$ (%)	${\mathrm{mAP}}_{50:95}$ (%)	Params (M)	GFLOPs	${\mathrm{mAP}}_{50}$ /GFLOP	${\mathrm{mAP}}_{50:95}$ /GFLOP	FPS
Faster R-CNN	40.3	54.1	46.2	22.1	41.3	83.4	0.55	0.26	–
RT-DETR-ResNet50	73.3	67.2	70.8	41.1	42.8	130.5	0.54	0.31	78.0
RT-DETR-ResNet101	67.9	67.3	69.7	40.1	67.3	232.4	0.30	0.17	60.1
YOLOv10-L	65.7	66.2	69.3	37.9	25.8	127.2	0.54	0.30	**104.8**
YOLOv12-X	**75.9**	**73.7**	**78.5**	41.4	59.1	199.9	0.39	0.21	49.0
RT-DETR-L	68.2	66.9	69.4	40.7	32.8	108.0	0.64	0.38	80.7
**SA-DETR (Ours)**	73.3	69.7	73.9	**42.7**	**21.0**	**57.8**	**1.28**	**0.74**	103.0

Note: Bold values indicate the best performance for each metric.

**Table 2:** Performance comparison on the PCB defect dataset over five independent stratified random splits (mean ± standard deviation).

Model	P (%)	R (%)	${\mathrm{mAP}}_{50}$ (%)	${\mathrm{mAP}}_{50:95}$ (%)
RT-DETR-L	87.0 ± 1.1	83.4 ± 2.0	85.7 ± 0.9	42.8 ± 1.3
SA-DETR	91.1 ± 2.4	83.8 ± 1.7	87.4 ± 1.2	44.4 ± 0.6

**Table 3:** Ablation results of the proposed modules on the NEU-DET dataset.

Model	P (%)	R (%)	${\mathrm{mAP}}_{50}$ (%)	${\mathrm{mAP}}_{50:95}$ (%)	Params (M)	GFLOPs	FPS
RT-DETR-L	68.2	66.9	69.4	40.7	32.8	108.0	80.7
O-EHGBlock	70.2	**75.4**	73.5	40.5	27.3	89.2	97.6
ASQS	74.2	70.5	72.4	41.3	32.8	108.0	97.0
StarC3	71.1	67.7	71.5	40.7	26.5	76.5	96.0
O-EHGB + ASQS	**75.5**	68.3	73.5	42.0	27.3	89.2	99.7
O-EHGB + StarC3	70.7	67.9	71.8	41.8	**21.0**	**57.8**	**107.5**
StarC3 + ASQS	71.0	68.5	72.0	41.8	26.5	76.5	95.7
**SA-DETR (Ours)**	73.3	69.7	**73.9**	**42.7**	**21.0**	**57.8**	103.0

Note: Bold values indicate the best performance for each metric.

**Table 4:** Sensitivity analysis of the channel ratio $r_{c}$ and structural weighting coefficient β on the NEU-DET dataset.

Setting	P (%)	R (%)	mAP_50_ (%)	mAP_50:95_ (%)	Params (M)	GFLOPs
$r_{c}=0.500$	68.5	**71.6**	72.5	42.5	**18.7**	**51.0**
$r_{c}=0.625$	72.5	64.1	71.0	40.9	19.7	54.0
$r_{c}=0.750$	**73.3**	69.7	**73.9**	**42.7**	21.0	57.8
$r_{c}=0.875$	70.9	69.0	71.7	40.6	22.5	62.2
$r_{c}=1.000$	70.3	64.6	70.5	40.6	24.2	67.2
β=0.1	71.6	67.0	70.2	41.0	21.0	57.8
β=0.2	70.2	66.7	70.6	40.5	21.0	57.8
β=0.3	73.3	**69.7**	**73.9**	**42.7**	21.0	57.8
β=0.4	**75.6**	66.3	70.7	40.3	21.0	57.8
β=0.5	73.4	67.7	72.3	41.7	21.0	57.8

Note: Bold values indicate the best performance for each metric.

## Data Availability

The datasets analyzed in this study are publicly available. The NEU-DET surface defect dataset is available from Northeastern University at https://faculty.neu.edu.cn/songkc/en/zdylm/263265/list/ (accessed on 13 September 2025). The PKU-Market-PCB dataset is available from the Open Lab on Human–Robot Interaction at Peking University at https://robotics.pkusz.edu.cn/resources/dataset/ (accessed on 13 September 2026). For the PCB experiments in this study, the 693 original defect images were used, excluding the corresponding rotated variants.

## References

[1] O’Donovan C., Giannetti C., Pleydell-Pearce C. (2024). Revolutionising the sustainability of steel manufacturing using computer vision. Procedia Comput. Sci..

[2] Tang B., Chen L., Sun W., Lin Z. (2023). Review of surface defect detection of steel products based on machine vision. IET Image Process..

[3] Girshick R. Fast r-cnn. Proceedings of the IEEE International Conference on Computer Vision.

[4] Liu W., Anguelov D., Erhan D., Szegedy C., Reed S., Fu C.Y., Berg A.C. (2016). SSD: Single Shot MultiBox Detector. Proceedings of the Computer Vision—ECCV 2016.

[5] Jiang P., Ergu D., Liu F., Cai Y., Ma B. (2022). A Review of Yolo algorithm developments. Procedia Comput. Sci..

[6] Mulajkar R., Yede S. YOLO version v1 to v8 comprehensive review. Proceedings of the 2024 International Conference on Inventive Computation Technologies (ICICT).

[7] Wen X., Yu Z., He Y., Wang Z., Chen X., Song K. (2026). Deep Learning-Based Steel Surface Defect Detection: A Survey. Coatings.

[8] Zhang H., Li M., Zhao K. (2024). A Feature Compensation Detection Network for Tiny Steel Surface Defects. Sensors.

[9] Touvron H., Cord M., Sablayrolles A., Synnaeve G., Jégou H. (2021). Going Deeper with Image Transformers. Proceedings of the IEEE/CVF International Conference on Computer Vision.

[10] Carion N., Massa F., Synnaeve G., Usunier N., Kirillov A., Zagoruyko S. (2020). End-to-End Object Detection with Transformers. Proceedings of the Computer Vision—ECCV 2020.

[11] Zhu X., Su W., Lu L., Li B., Wang X., Dai J. (2020). Deformable DETR: Deformable transformers for end-to-end object detection. arXiv.

[12] Liu S., Li F., Zhang H., Yang X., Qi X., Su H., Zhu J., Zhang L. (2022). Dab-detr: Dynamic anchor boxes are better queries for detr. arXiv.

[13] Zhang H., Li F., Liu S., Zhang L., Su H., Zhu J., Ni L.M., Shum H.-Y. (2022). DINO: DETR with improved denoising anchor boxes for end-to-end object detection. arXiv.

[14] Zhao Y., Lv W., Xu S., Wei J., Wang G., Dang Q., Liu Y., Chen J. (2024). DETRs Beat YOLOs on Real-Time Object Detection. Proceedings of the IEEE/CVF Conference on Computer Vision and Pattern Recognition.

[15] Wan H., Wu S., Chen X., Ren P. (2025). An Improved RT-DETR Model for High-Performance Steel Surface Defect Detection. Proceedings of the 2025 IEEE 6th International Conference on Pattern Recognition, Information and Artificial Intelligence.

[16] Ayon S.T.K., Siraj F.M., Uddin J. (2025). Steel surface defect detection using learnable memory vision transformer. Comput. Mater. Contin..

[17] Zhang X., Chai Y., Hu Y., Shang Y., Tao Z., Ma Y. Steel Surface Defect Detection Algorithm Based on Improved RT-DETR. Proceedings of the 11th International Conference on Computer and Communications (ICCC).

[18] Liu Y., Zhang Y., Wang Y., Zhang Y., Tian J., Shi Z., Fan J., He Z. SAP-DETR: Bridging the Gap Between Salient Points and Queries-Based Transformer Detector for Fast Model Convergency. Proceedings of the 2023 IEEE/CVF Conference on Computer Vision and Pattern Recognition (CVPR).

[19] Hou X., Liu M., Zhang S., Wei P., Chen B. Salience DETR: Enhancing Detection Transformer with Hierarchical Salience Filtering Refinement. Proceedings of the 2024 IEEE/CVF Conference on Computer Vision and Pattern Recognition (CVPR).

[20] Li T., Song W., Chen N., Gao F., Wu S., Song C., Qi W., Lin Y., Yin R. (2025). Lightweight RT-DETR for Steel Surface Defect Detection. Proceedings of the 2025 International Symposium on Machine Learning and Social Computing.

[21] Wang R., Ma N., Luo Q., Zhou X., Gu S. (2025). DCE-YOLOv8n: A lightweight attention-enhanced model for weld surface defect detection in low-light scenes. Eng. Res. Express.

[22] Yu J., Cheng X., Li Q. (2021). Surface defect detection of steel strips based on anchor-free network with channel attention and bidirectional feature fusion. IEEE Trans. Instrum. Meas..

[23] Ding H., Chen J., Ye H., Chen Y. (2025). Yolo-mad: Multi-scale geometric structure feature extraction and fusion for steel surface defect detection. Appl. Sci..

[24] Chen C., Chen M., Lee H., Li Y., Yu J. (2025). A Steel Surface Defect Detection Method Based on Lightweight Convolution Optimization. arXiv.

[25] Zhang Z., Guoyun Z., Ding P., He J., Zhang J., Chongyang Z. (2025). ELS-YOLO: Efficient lightweight YOLO for steel surface defect detection. Electronics.

[26] Zhou H., Zou H., Hu G. (2025). An efficient and lightweight algorithm for detecting surface defects of steel based on SCCI-YOLO. Sci. Rep..

[27] Xia Z., Huang Z., Gu J., Wang W. (2025). Surface Defect Detector Based on Deformable Convolution and Lightweight Multi-Scale Attention. Concurr. Comput. Pract. Exp..

[28] Zhao W., Song K., Wang Y., Liang S., Yan Y. (2023). FaNet: Feature-aware network for few shot classification of strip steel surface defects. Measurement.

[29] Huang W., Wei P. (2019). A PCB Dataset for Defects Detection and Classification. arXiv.

[30] Lin T.Y., Maire M., Belongie S., Hays J., Perona P., Ramanan D., Dollár P., Zitnick C.L. (2014). Microsoft coco: Common objects in context. Proceedings of the European Conference on Computer Vision.

[31] Wang A., Chen H., Liu L., Chen K., Lin Z., Han J., Ding G. (2024). YOLOv10: Real-Time End-to-End Object Detection. Adv. Neural Inf. Process. Syst..

[32] Tian Y., Ye Q., Doermann D. (2025). YOLOv12: Attention-Centric Real-Time Object Detectors. Adv. Neural Inf. Process. Syst..

