# Correction: Liang, P.; Wu, X. SA-DETR: Lightweight Structure-Aware Defect Detection Model for Sustainable Low-Carbon Steel Manufacturing Based on RT-DETR-L. *Sensors* 2026, *26*, 6075

**DOI:** 10.3390/s26216789

**Published:** 2026-10-27

**Authors:** Peifeng Liang, Xinyue Wu

**Affiliations:** School of Computer Engineering, Jiangsu University of Technology, Changzhou 213000, China; 2024655156@smail.jsut.edu.cn

**References Correction**

In the original publication [1], References 1, 7, 16, 17, 21, and 26 could not be located; the corrected References 1, 7, 16, 17, 21, and 26 are listed below.

1. O’Donovan, C.; Giannetti, C.; Pleydell-Pearce, C. Revolutionising the sustainability of steel manufacturing using computer vision. *Procedia Comput. Sci.* **2024**, *232*, 1729–1738. https://doi.org/10.1016/j.procs.2024.01.171.

7. Wen, X.; Yu, Z.; He, Y.; Wang, Z.; Chen, X.; Song, K. Deep Learning-Based Steel Surface Defect Detection: A Survey. *Coatings* **2026**, *16*, 1040. https://doi.org/10.3390/coatings16091040.

16. Ayon, S.T.K.; Siraj, F.M.; Uddin, J. Steel surface defect detection using learnable memory vision transformer. *Comput. Mater. Contin.* **2025**, *82*, 499–520. https://doi.org/10.32604/cmc.2025.058361.

17. Zhang, X.; Chai, Y.; Hu, Y.; Shang, Y.; Tao, Z.; Ma, Y. Steel Surface Defect Detection Algorithm Based on Improved RT-DETR. In Proceedings of the 11th International Conference on Computer and Communications (ICCC), Chengdu, China, 12–15 December 2025; pp. 894–900. https://doi.org/10.1109/ICCC68654.2025.11437934.

21. Wang, R.; Ma, N.; Luo, Q.; Zhou, X.; Gu, S. DCE-YOLOv8n: A lightweight attention-enhanced model for weld surface defect detection in low-light scenes. *Eng. Res. Express* **2025**, *7*, 035235. https://doi.org/10.1088/2631-8695/adf174.

26. Zhou, H.; Zou, H.; Hu, G. An efficient and lightweight algorithm for detecting surface defects of steel based on SCCI-YOLO. *Sci. Rep.* **2025**, *15*, 20154. https://doi.org/10.1038/s41598-025-20154-y.

The authors state that the scientific conclusions are unaffected. This correction was approved by the Academic Editor. The original publication has also been updated.
